# A qualitative study on the use of long-lasting insecticidal nets (LLINs) for the prevention of malaria in the Peruvian Amazon

**DOI:** 10.1186/s12936-019-2937-1

**Published:** 2019-09-02

**Authors:** Mia Iyer, John Skelton, Giles de Wildt, Graziela Meza

**Affiliations:** 1 Department of Population and Health Science, University of Birmingham, Birmingham, UK; 2 Facultad de Medicina Humana, Universidad Nacional de la Amazonia Peruana, Iquitos, Peru

**Keywords:** Qualitative, Malaria, Malaria prevention, Bed net, LLIN, Peru

## Abstract

**Background:**

Malaria is a huge global health burden due to its mortality, morbidity and cost to economies. It is necessary to eliminate the disease in all countries where possible to achieve the World Health Organization target of > 90% reduction by 2030. Successful previous campaigns suggest elimination is feasible in Peru. However, the incidence has recently been rising, focalized to the region of Loreto. Currently, the distribution of long-lasting insecticide-treated nets (LLINs) is a major part of Peru’s malaria control strategy, however these may be having a limited effect in Loreto, because of the recent behavioural adaption of the mosquito vector, *Anopheles darlingi*, to earlier biting times, as well as local perceptions and practices towards LLINs. It was, therefore, necessary to investigate how perceptions, practices and lifestyle factors affect the efficacy of LLINs in Loreto.

**Methods:**

Qualitative research was carried out in 5 rural communities along the Iquitos-Nauta Road in Loreto, which have increased exposure and have received nets in a distribution scheme prior to the study. Twenty semi-structured interviews as well as observations of the bed nets were conducted in participants’ homes, using a topic guide. Thematic content analysis was used to produce the findings.

**Results:**

All participants viewed malaria prevention as a high priority, and the use of bed nets was deeply embedded in the culture. They expressed preference for LLINs over traditional-type nets. However there were too few LLINs distributed, participants did not maintain the nets correctly, washed them too frequently and did not repair holes. The earlier mosquito biting times were also problematic. Additionally, poor housing construction and proximity to mosquito breeding sites further increased transmission.

**Conclusion:**

The positive findings in attitudes of the respondents can be used to improve malaria control in these communities. Interventions providing education on effective LLIN use should be implemented. A change in strategy away from vector control methods is also necessary, as these do not provide long-term protection due to the adaptability of *An. darlingi*. Interventions focusing on parasite control are recommended, and socio-economic factors which increase malaria risk should be addressed.

## Background

Malaria, a vector borne parasitic disease transmitted by the female *Anopheles* mosquito, is an illness which can be fatal without appropriate treatment. It is the seventh leading cause of mortality in lower income countries [1], and is responsible for 5% of under-five child deaths globally [2]. It is recognized to be a huge global health burden and although the disease is both preventable and curable, it is still endemic in 91 countries and had 216 million cases in 2016 [3]. There have been consistent efforts to tackle the disease globally, with campaigns reaching $2.7 billion in 2016 [3]. This number is expected to rise to $6.5 billion by 2020. The cost of campaigns, as well as the lives lost due to the disease, creates a huge loss to economies. Elimination is, therefore, imperative. The Global Technical Strategy for elimination of malaria, developed by the World Health Organization (WHO), aims to reduce malaria by > 90% by 2030 as compared with 2016 [4]. To achieve this target, swift action is necessary to eliminate malaria in countries where it is possible.

Peru currently has the second highest malaria prevalence in South America [5], but elimination of malaria in Peru is thought to be possible due to the success of previous national control efforts, which decreased malaria prevalence by 90% between 1998 and 2011 [6]. However, despite the success of this campaign, the incidence of malaria has been rising since 2011, which indicates that Peru should be the focus of investigation [3].

*Plasmodium vivax* is the dominant cause of malaria infection in Peru, which makes elimination more difficult due to the nature of *P. vivax*, which allows transmission of infection even in asymptomatic individuals due to activating dormant hypnozoites in the liver [7]. Additionally, the incidence of cases caused by *Plasmodium falciparum* has been increasing since the 1980s and now causes 16.9% of cases [8, 9]. This is also a cause of concern as *P. falciparum* malaria carries a higher risk of mortality. Currently 95% of the malaria prevalence in Peru is located in Loreto, which is also the location of 99.6% of Peru’s *P. falciparum* cases [9, 10]. Due to its rainy weather and high humidity, the Loreto region has many swamps and slow-flowing streams which act as malaria ‘hotspots’ as they provide ideal conditions for *Anopheles darlingi* to breed [11]. This suggests that Loreto should be the focus of control efforts aimed at elimination.

It should be noted that funding for malaria control in Peru comes primarily from the Peruvian government, which has spent only $0.1 per person at risk of malaria between 2014 and 2016, the third lowest amount for all countries in the WHO Americas region and contrasts hugely, with countries such as Suriname which spends $1.8 per person at risk of malaria [3].

A major component of Peru’s current malaria control strategy is the distribution of long-lasting insecticidal nets (LLINs) to the population. LLINs are recommended by the WHO as an effective vector control method, with the ability to reduce malaria incidence by 50% [12]. However, in Peru LLINs may not be as effective in reducing transmission. There has been increasing evidence reporting behavioural changes in *An. darlingi*, which is the main malaria vector in Loreto, with an increased exophilly and earlier biting times, as an adaptation to the use of vector control methods [13, 14]. Its peak biting time now starts from early evening, with one study specifically citing 6 p.m. as the biting time, which is when most people are unprotected by their bed nets [13, 15, 16]. This change has also been perceived in a recent qualitative study conducted in Iquitos [17], where participants to the study viewed bed nets as inadequate protection. Qualitative research in rural communities in Loreto has often shown that people do not use LLINs, preferring traditional *tocuyo* nets [18, 19]. These nets are made from muslin-like cotton and are untreated, which make them half as effective as treated nets [20]. People from these communities preferred these nets since the smaller mesh size of the fabric offers more privacy than LLINs. The transparency of LLINs allow them to function less effectively as room dividers and as protection from spirits, which is regarded as one purpose of bed nets in these communities [18]. Also, the larger mesh size of the LLIN allows for less warmth when sleeping under the nets in comparison to the *tocuyo* nets, and many have cited this as a reason to use the latter, even though they have treated nets [18, 19]. Additionally, those who use treated nets have been reported to wash them more frequently than every 3 months as recommended by the WHO [19, 21, 22]. This time-frame is recommended as any more frequent washings can reduce the levels of insecticide within the nets and limit its protection [22]. One study in particular found that participants washed their nets bi-weekly [19]. Qualitative evidence from Iquitos has also reported that many individuals do not consider personal prevention to be a priority, due to the frequency of the disease and the view that malaria is inevitable [17].

It is, therefore, likely that behavioural factors which affect the efficacy of LLINs contribute to the increasing malaria incidence in Peru. However, despite previous qualitative research on bed net preferences in rural communities [18], and vector control methods in urban settings [17], there is no recent qualitative data on how perceptions and practices towards LLINs specifically is affecting their efficacy in rural communities, which have a higher risk of malaria [23]. It is imperative to conduct qualitative research investigating the perceptions and practices relating to LLIN use in rural communities.

## Methods

### Aims and objectives

The aim of this study was to conduct qualitative research in rural villages in Loreto in order to (1) investigate the level of knowledge about malaria and the use of LLINs, (2) understand how this knowledge influences perceptions about LLINs, (3) understand how these perceptions interact within communities to influence behaviours which affect the use of LLINs.

### Setting

Interviews were conducted in 5 villages along the Iquitos-Naura road in Loreto, in January and February 2018. These communities are hyper-endemic for malaria [8], as they are rural and situated near the upper Amazon basin which increases their exposure to *An. darlingi* [23]. The study was conducted during the region’s rainy season (occurring from January to June) as this is when these communities are most at risk of malaria [24]. Communities along this road had also received LLINs in a distribution scheme conducted in 2017 by the Rotary club, which distributed Permanet 3.0 to communities [25], making them ideal areas for qualitative research on this topic.

**Fig. 1:**
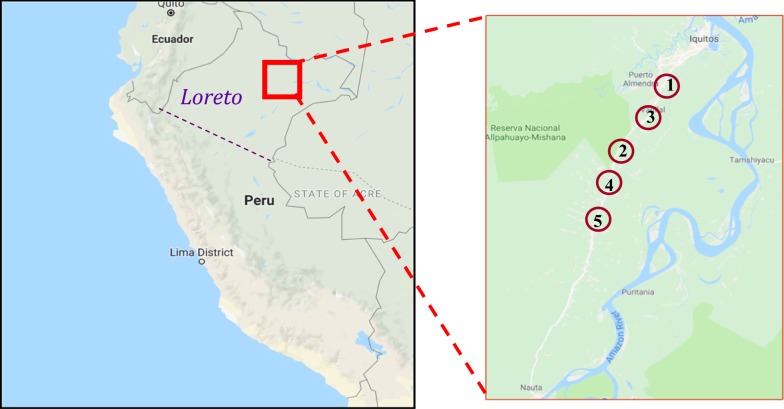
A map of where the Loreto region is located in Peru, and where each village included in the study is located

A minimum of five villages were chosen for the study. This enabled each village to have three or more households participating in the study, to allow the collection of data which was more representative of the entire village population than if fewer households were recruited per village. Villages that were chosen were all close to the Iquitos-Nauta road as difficulties in obtaining transported prevented the research team from reaching villages which were located far from the road (Fig. 1).

### Sample

To be included in the study, participants had to be aged 18+, a resident in the selected village and able to speak either English or Spanish fluently. The exclusion criteria was refusal to show their bed net to the researchers and the inability to consent. Participants were aged between 18 and 72. Included in the study were 6 men and 14 women.

### Sampling strategy

Convenience sampling was felt to be the most appropriate strategy for selecting participants, as the wide inclusion criteria signified that no specific demographic group was being targeted for the data [26]. For this reason, purposive sampling was felt to be unnecessary [27]. Additionally, convenience sampling offered more easily available access to data and was the most straightforward to implement.

To select participants, the centre point of the village was located and a direction was chosen at random. Then the research team went door-to-door in the direction chosen to recruit the participants. This method was used in order to reduce selection bias, and the use of more-than one direction was deemed unnecessary as there has not been any reported difference in the malaria prevalence between houses in the same villages in this region. Potential participants were asked about their availability before being asked to take part in the study. Participants were not allowed to self-select or recruit other participants.

The initial study design intended to include equal numbers of men and women, however during the study there it was discovered that women in these communities were more aware of the reasoning behind the family’s bed net practices than men. For example, two male participants required female family members to answer questions on their behalf regarding the reasoning behind the family’s bed net practices, and one male refused to participate in an interview as he claimed that his mother was responsible for decisions regarding bed net practices in the household. After this discovery, in those households which had agreed to participate in the study, it was the females who were chosen to be interviewed instead of the males as they were able to offer more information during the interview. This affected the study by providing more female participants than males and, therefore, went against the initial study design, however it allowed more information to be gathered during the interview process.

### Study design

A qualitative approach was taken due to its ability to explore human behaviour in depth, and generate ideas which are transferrable [28]. Face-to-face interviews were used to achieve in depth exploration of the topic, and to allow the interviewer insight into verbal and non-verbal forms of communication [29]. Interviews were chosen instead of focus groups as the study is concerned with the behaviours of individuals towards bed nets, and bed net use is an individual activity. Also, social desirability bias is more likely to occur in a focus group: this can cause participants to give inaccurate answers to questions regarding bed net habits [30]. The interviews were semi-structured, using open ended questions, to facilitate the exploration of key topics as well as free expression of ideas from the participant [30]. Additionally, observations of the participants’ bed nets were conducted. This was to check if the participant had been using the net, and to see how it had been maintained.

### Data collection

20 participants were recruited and interviews were conducted in five villages as follows: 5 in Zungarococha, 4 in El Dorado, 4 in Rosa Mystica, 3 in Pujil and 4 in Ex Petrolieroz zone 1.

The aim of the study design was to recruit at least 20 participants as previous research in this area has suggested that analytical saturation would be reached before this point [17]. As no new themes began to emerge from the data after the 16th interview, 20 participants was deemed sufficient as saturation had been reached [28]. If this had not been the case, a further 10 participants would have been recruited however this was not necessary.

The interviews were conducted in the participants’ homes, which facilitated observation of the bed nets. Interviews took place on the same day as the recruitment. The purpose of the study was explained to the participant in Spanish, which was the local language, to ensure informed consent. They were then given the participation sheet, which had been translated into Spanish before obtaining written consent for the interview and the observation. Each interview was preceded with the participant being asked to confirm their age and demographics. Interviews were conducted using a translator who was fluent in both Spanish and English. The translator was recruited through a language school (Universidad Nacional de la Amazonía Peruana) in Iquitos, and had conducted qualitative interviews 1 year previous to the time of the study. The translator was bound to maintaining confidentiality with respect to the study.

The semi-structured interviews were based on a topic guide, which was developed by reviewing previous research that was relevant to the topic. This allowed exploration of a wide range of subjects during the interview, whilst also being flexible, to allow exploration of new ideas presented by the participant. The topic guide was piloted in the first interview, and edited during the data collection process in response to emerging themes. It was also translated into Spanish to allow the translator to facilitate the interview.

During the interview, the researcher would ask a question from the topic guide in English, which was translated into Spanish by the translator for the understanding of the participants as none of the participants were fluent in English. The participant would answer in Spanish, and their answer would be translated into English for the understanding of the researcher. This would then allow the researcher to ask more questions in order to understand the participants bed net practices. The interview was not conducted fully in Spanish despite this being the local language, as the researcher was not fluent in Spanish however it was necessary for the researcher to understand the participants’ responses during each interview in order to direct the interview.

After each interview, the bed net observations took place. The participant showed the researcher and the translator each bed net in the house, and the researcher was able to observe how the bed net had been set up and observe its current state. The participant was also asked questions about how they maintained their bed nets.

Each interview and observation lasted between 20 and 60 min, and was recorded on a device, which was encrypted for data protection purposes. Each participant provided written consent for this to be done. A verbatim transcription of the English translation was produced from each recording. While the audiorecording in Spanish was retained and used for verification, no attempt was made to create a detailed word-by-word transcript of the original Spanish. However, the English transcript, taken from the contemporaneous oral translation offered to the researcher was transcribed in detail. 10% of these transcripts were checked for content against the original Spanish responses by JRS, who is a linguist.

Field notes were also recorded to contextualize the data and provide reflections on each interview.

Participant data was later anonymized, with each participant being allocated a participant number.

Difficulties in re-contacting participants prohibited respondent validation, as participants were not willing to give out their mobile numbers and there would have been difficulties in contacting them from the UK.

### Analysis

Thematic content analysis was used, as it is a flexible approach which allows the understanding of underlying patterns of behaviour. This was done using the Braun and Clarke 6 step inductive analysis technique [31]:

1. Familiarization with and immersion in the data.
2. Generation of initial codes from patterns in the data.
3. Understanding the relationship between codes to form initial themes.
4. Reviewing themes to ensure mutual exclusivity and that they are reflective of the data set.
5. Defining themes by analysing the data contained within each theme.
6. Producing a report which contains the final analysis.

Transcripts were analysed by MI using an iterative, data driven approach. Clean, verbatim transcripts were produced to improve narrative flow, as the translator often spoke in somewhat hesitant English [32]. MI repeatedly read the transcripts to immerse herself within the data. They were then coded and compared using the constant comparison method [33]. 25 initial codes were generated. The codes were then analysed, using the field notes, to understand the context behind them and how they interacted with each other.

Additional codes were created to reflect ideas generated from the data which were apparent but not specifically stated. All codes were then collated to form a complete understanding of the behaviours which influence LLIN effectiveness, and were then analysed to form themes. This was done by creating a thematic map, and this process was repeated six times.

All transcripts were analysed independently by JS. Analyses were then compared to generate the final themes.

### Ethics

Ethical approval was granted by the Institutional Research Ethics Committee at the Department of Health, Loreto and the Population Sciences and Humanities Research Ethics Committee at the University of Birmingham. Data handling was in accordance with the Data Protection Act 1988 [34]. Interviews were recorded on an encrypted recording device and all interview data was stored on a password protected laptop. Participants were given an individual identification number, so there was no personal identifiable information attached to the data.

## Results

5 themes emerged after analysing the data:

1. Knowledge of malaria.
2. Culture of bed net use.
3. Factors which were limiting the protection of LLINs.
4. Socio-economic factors further increasing the malaria risk.
5. Attitudes to future interventions.

### Knowledge of malaria

All participants knew that malaria was an illness which was transmitted by mosquitoes. Three participants referred to malaria as a *“virus”* [P07] [P13] [P20], however no-one specifically used the word ‘parasite’. Six participants knew that malaria had different types [P02] [P03] [P04] [P11] [P17] [P18], with five of them naming “*falciparum*” and “*vivax*” as types and one participant referring to malaria as having “*haemorrhagic*” and “*passive*” types [P18]. Two participants were aware that falciparum was “*stronger*” than vivax [P02] [P11], however one participant thought that malaria vivax was more “*dangerous*” [P04] and another participant was confused about which type of malaria was worse:

*“And I know a type, falciparum, it is very strong……I think vivax is more dangerous, and stronger than the falciparum.”* [P03]

One participant referenced malaria affecting red blood cells:

*“It is an illness that affects your red blood cell.”* [P17]

Three participants were aware that living in highly vegetated areas increased their exposure [P06] [P11] [P12]. For example P06 said:

*“You can have malaria when you live near the trees, near to the jungle.”* [P06]

All participants except for one [P16] had experience of either themselves or close family members having the disease, and all participants had knowledge of the symptoms of malaria. Three participants were also able to describe the treatment for malaria [P02] [P06] [P13], with one of whom [P13] naming primaquine and chloroquine, which is the correct treatment for *P. vivax* in this region.

All participants viewed malaria as a serious illness. This view was attributed the risk of mortality by seven participants, as well as the “*bad symptoms*” [P03] [P15]. One participant also commended that she was “*afraid*” of malaria [P18]. Prevention was considered a high priority because of this:

“*A dangerous disease. If we have awareness, if everyone has awareness, we can prevent this disease.”* [P07].

Most participants understood the importance of preventing mosquito breeding sites, for example one participant said: “*The objects where we put water in, they should be closed covered*” [P01].

There were some misconceptions about the disease. Two participants thought that drinking water which had been boiled was preventative [P07] [P13] and three participants thought that having a clean house prevented malaria [P19] [P06] [P07].

*“To prevent this disease you should keep your house clean”.* [P06]

*“I am very careful to prevent this disease. I protect my children by boiling water and sleeping with mosquito bed nets.”* [P07]

However, the majority of the participants considered contaminated water to be the source of infection, which signifies that the causal relationship is known but not well understood.

*“Keep clean water…This is a way to prevent this disease.” [P11]*

*“Malaria is a kind of Virus. It comes when you don’t treat your water.”* [P13]

Additionally, some participants did not understand the difference between malaria and dengue. One participant said that malaria was caused by “*a big mosquito called dengue*” [P08].

Five participants also mentioned the use of traditional herbal treatments as effective for preventing and treating malaria [P04] [P05] [P08] [P10] [P15], and two participants specifically referred to drinking the juice of a fruit called ‘*toronja*’ to prevent and treat malaria. This is a citrus fruit which closely resembles a grapefruit, and grows locally to this region [P05] [P10].

*“We decided to not take pills, to not take medicines from the hospital, from the doctors. So we decided to use natural medicines…when I had malaria, my daughter told me that I had to drink the juice of the ‘toronja’. You have to mash the pulp of the toronja and drink this for three days.”* [P10].

Teaching on malaria was found to be inconsistent. Six participants had learnt about the disease from nurses who educated people in the community about common diseases [P01] [P06] [P11] [P13] [P18] [P20], and three had learnt through school teaching programmes [P02] [P07] [P15]. However, most participants did not know about malaria when they were young, and seven participants only learnt about the disease when they or a family member were being treated [P03] [P04] [P05] [P08] [P14] [P16] [P19].

*“When I was at school, nurses went there to teach students about malaria.”* [P02]

*“I knew about malaria from each time that I went to the hospital”* [P19].

Two participants said that malaria was commonly known by another name, “*Paludismo*” [P19] [P07]. (This is a common term in the Spanish-speaking world). One of these participants reported that when she was young, people only knew this name and not ‘malaria’. [P19]

### Culture of bed nets

All participants slept under either traditional or LLINs every night. This was largely due to the cultural use of nets, and all participants except for one reported sleeping under bed nets since they were very young.

“*I used bed nets from when I was born”* [P13].

However one participant reported that before her child had malaria, she would only sleep under a bed net during the rainy season, as this was the custom in the community which she used to live in:

“*When I lived at San Martin I just used mosquito bed nets when it rained So my children used to sleep without mosquito bed nets. For that reason when I came here with my family, I didn’t use mosquito bed nets. But when my child had malaria I started to use bed nets even when it was hot.”* [P07]

Participants liked sleeping under nets because of the protection they offer against mosquitoes:

“*I feel protected because mosquitoes cannot come into the bed”* [P15].

All participants had slept under traditional untreated nets, the majority of which were made from ‘*tocuyo*’, before they started to sleep under LLINs. These were purchased in Iquitos, which is the nearest city.

“*Before this, getting nets from the Rotary club, I had to buy my bed nets…*

*…They were made of ‘tocuyo’”* [P17].

All participants preferred LLINs to traditional nets. This was due to participants feeling more protected from mosquitoes whilst underneath them, and liking how they felt cooler whilst sleeping under it.

“*I prefer the mosquito bed net that we received by the program because the texture feels more comfortable, because when we are asleep we can feel the air flow easily, and the other common traditional bed net is not the same”* [P02].

“*Of course I prefer the mosquito bed net with insecticide, because it kills the mosquitoes. The traditional ones just protect from mosquitoes, but they don’t kill them. With the new mosquito bed nets with insecticide, the texture is fresh and we can feel the air flow more”* [P05].

Participants who did not have LLINs wanted to have one [P07] [P15]:

“*If I had the opportunity to get a mosquito bed net with insecticide, I would prefer to sleep under that*” [P07].

One participant remarked that the LLIN offered less privacy than the traditional net [P17], however this was something which she had become used to. Another participant had the same view regarding the irritant effects of the insecticide in LLINs:

“*The first weeks, when you start to use the bed nets with insecticide, you get a rash. But it’s normal, so after a few days it passes”* [P18].

All participants reported that they would repair holes in the net if they occurred. The majority of them reported that they tucked the net in during use. However, four participants reported that they would lie the bottom of the net on the bed, so it was touching the bed all around during use [P04] [P11] [P14] [P17]. For two participants this was due to them not owning mattresses and having to sleep on a sheet which had been laid on wooden slats, making it difficult to tuck the net in [P11] [P14]. Two participants said that they would use clothes or other items to weigh down parts of the bed net which were lying on the bed, to prevent it from moving [P04] [P17]. All participants knew the correct procedure of hanging the net after receiving it to distribute the insecticide.

Additionally, most participants used bed nets as their only method of preventing malaria, except for three who used “*incense*” [P13] [P15] [P17] and one participant who sprayed the walls and the floor with insecticide [P01].

### Limited protection of LLINs

There were three reasons why the protection offered by LLINs might in fact be limited. These were (i) participants not making use of the net, despite the LLIN distribution scheme, either because they preferred to use the traditional net, or did not receive an LLIN, (ii) incorrect use of the LLIN, (iii) participants not using the net during mosquito biting times.

#### (i) Not making use of the LLIN

Six participants reported use of the traditional net within their household. For three of them, this was due to them not receiving a net in the distribution scheme. One participant had been unaware that the scheme had been taking place, and the remaining two had been unable to receive them as there were not enough nets to supply every member of the community. This was because one participant had arrived to the collection point late in the day, after all of the nets had been distributed [P07], and the other participant lived in a village where every family could only receive two bed nets, which was not enough to cover every household member [P13]:

*“It was very late when I noticed that my neighbours were receiving mosquito bed nets in the mini hospital. And when I went there, it was late and there weren’t any more mosquito bed nets for me.” [P07].*

Two participants reported use of the traditional net due to their children reacting to the irritant effects of the insecticide in the LLINs:

*“It has too much poison and I don’t want to use it… because of the baby… because he can get a rash”* [P02].

*“I only know that when my son starts to sleep under the bed net given by the Rotary club, he has an allergy. For that reason my son doesn’t want to sleep with the new one. So he still is sleeping under the traditional”* [P16].

One participant was also waiting for an old traditional net to be worn before using an LLIN [P03].

#### (ii) Incorrect use of the LLIN

Despite the majority of participants knowing the correct maintenance procedure of washing their bed nets more frequently than every 3 months, as recommended by the WHO [22], eight participants washed their nets more frequently than this:

“*Yes, I wash it every week. With water, soap and detergent”* [P05].

“*I wash them with detergent…maybe once per month. Or maybe just once per two months”* [P09].

Holes were also found in the nets of five participants during observations [P02] [P08] [P09] [P10] [P13], despite all participants reporting that they would normally repair holes. This indicates delays in repair, or possible reporting bias.

Additionally, five participants were using the nets above capacity. The distributed LLINs allow a maximum of two people to sleep underneath, however it was found during four interviews that three people were sleeping under a net [P09] [P10] [P17] [P18], and in one household there were four family members sleeping under one LLIN [P12].

“*There are four of us and all of us sleep in the same bed*” [P12].

#### (iii) Dissonance between mosquito biting time and LLIN use

Four participants reported that mosquitoes had started to bite from 6 p.m. onwards, however none of the participants reported that they were using a bed net by this time:

*“From six o’clock the mosquitoes come and they start to bite people” [P04].*

No participants were under their bed nets before 7 p.m., and only two were under their nets between seven and eight p.m. Several participants were under their nets from 8 p.m. onwards, which leaves a 2 h unprotected window:

*“There are too many mosquitoes at night. From 6…but me, I sleep at 9 or 10 P.M.” [P07].*

*“All of us sleep from 8P.M.”* [P09].

Lifestyle appeared to be the main reason why participants were not under their bed nets by six:

“*My work finishes at 6. Then I have a shower and then I go to my son’s house and watch TV until 9.”* [P11]

*“We sleep depending on my baby, because most of the time my baby goes to sleep between 10, 11.*

*Before we go to sleep we are here* [in the lounge area]*, waiting for the baby to fall sleep.”* [P17]

### Socio economic factors increasing risk

There were four factors which are known to increase the risk of malaria in the communities, therefore counteracting the protective effects of the LLIN distribution scheme:

#### (i) Poorly constructed housing

All houses in which interviews took place had open eaves. The majority of them had walls made from wooden planks which had been joined together, but had gaps in between. No houses were observed to have window or door screens. No houses had air conditioning, and only one house had a fan. Participants frequently opened their windows during the day and early evening due to the heat, which facilitated entry of mosquitoes.

#### (ii) Increased number of mosquito breeding sites

In three villages where interviews were conducted (Zungarococha, Rosa Mystica and Ex Petrolieros Zone 1) several pools of stagnant water were observed on the streets. In Rosa Mystica there was a lake which appeared polluted, and a pond which was filled with litter. None of these mosquito breeding sites had been cleaned by the authorities. One participant expressed dissatisfaction with this:

“*I think that the government has to do a clean of the public areas because there is too much garbage. Too many mosquitoes grow in those places.”* [P02].

#### (iii) Remoteness

The location of one village (Rosa Mystica) prohibited the net distribution scheme from occurring in the community. Community members had to travel to a nearby village to receive their nets, and were only able to receive two nets per household. This caused one participant from this village to not have a LLIN [P13], and two participants from here were using LLINs above capacity [P10] [P12]:

“*They told the people to go to ‘Barijal’* [the nearby village] *to receive the mosquito bed net, but unfortunately only some people from Rosa Mystica received the mosquito bed nets. Because people from Barijal got them first.”* [P10].

The remoteness of the communities also made it difficult for many participants to purchase bed nets before receiving a distributed net, as it required traveling to Iquitos:

“*It was difficult* [to buy bed nets] *because I had to travel for a long time to buy it”* [P14].

One participant also said that their nearest health facilities were not easily accessible, which made it difficult for them to seek prompt treatment when unwell. This caused the participant to think that *“natural medicines”* were necessary:

“*The nearest hospital from here is far. So people have to use natural medicines….for example, if you have the disease at night there is no transportation to get to the hospital”* [P08].

#### (iv) Cost of prevention

Prior to the distribution scheme, participants found the cost of traveling to Iquitos to buy nets expensive. They would often buy the cheapest nets, which were untreated:

“*It was difficult for me to go to the city and buy my bed nets because often there was not enough money for transportation to get bed nets. So you have to work a lot to save money to go there”* [P18].

“*I buy the cheapest. But the most expensive are better because they last longer.”* [P07]

Additionally, none of the participants used topical repellents. This is because they found them too expensive:

“*I don’t have enough resources to buy skin repellents”* [P17]

*“People here, they don’t have the possibility* [of buying repellants] *because the salary that they have is very low. For that reason they cannot buy repellents.”* [P20]

### Attitudes to future intervention

All participants thought that additional interventions were necessary to prevent malaria.

Thirteen participants reported that they found distributions schemes to be beneficial, and several participants requested for them to continue:

“*I am very thankful to have bed nets with insecticide given by private or public organizations”* [P12]

“*I feel that the government helps us, it helps the village because we receive mosquito bed nets to prevent malaria.”* [P08]

Two other participants asked for other interventions regarding prevention. One participant asked for a vaccine [P04] and another asked for more research to develop insecticides, which are more effective:

“*The government must run good projects, for example to search or research for insecticides which kill more effectively.”* [P12]

Three participants requested for the government to improve public cleanliness [P02] [P04] [P10]:

“*We would like the government to do a programme to bring water, but clean. The water that we drink here is polluted.”* [P10]

Two participants wanted improved health education about malaria [P06] [P09]:

*“So, me and my family don’t receive information about malaria…but I want to get information about it.”* [P09]

Seven participants asked for more fumigation as they regarded this as effective malaria prevention. As it became clear that participants understood indoor residual spraying (IRS, a method used to prevent malaria) to be a form of fumigation, it was understood that participants were requesting more frequent IRS, which is currently performed once or twice per year in each of the communities:

“*So I recommend to the government more fumigations in a year.”* [P03].

## Discussion

Participants demonstrated good basic knowledge of malaria. This indicates that previous education programmes have achieved a measure of success in increasing awareness of the disease, and that future interventions may, therefore, also be effective, as it is already known that increased knowledge about an illness makes prevention methods more effective [35].

Additionally, participants considered personal prevention against malaria to be a high priority. This contrasts with qualitative evidence from only a year ago showing that people in Iquitos did not consider personal prevention to be a priority because of the view that malaria was inevitable [17]. However despite the disease also being common in the rural communities along the Iquitos-Nauta road, here it has led to the view of malaria being a serious illness, which requires personal prevention. This contrasting view between rural and urban settings suggests that people are more conscientious about prevention in areas which are higher risk for malaria.

However, misconceptions about drinking boiled water and cleaning the house being effective prevention methods exist despite numerous educational efforts. These misconceptions have been identified in previous qualitative research in this region [17, 36], and demonstrate a persisting problem. It is possible that participants may not understand the difference between malaria prevention and prevention of other water-borne diseases, such as typhoid, as it was clear that many participants may not have understood the difference between malaria and dengue. However, it is highly important that this lack of understanding is corrected as evidence has shown that misconceptions about malaria have a negative impact on health-seeking behaviour [37].

The teaching about malaria is also inconsistent between communities. Some participants had learnt about the disease through teaching by district nurses or school teaching programmes. However many were only taught about malaria through experience with the illness. The latter can be associated with increasing a person’s risk of malaria as they are often unaware of the disease before they are first infected, and could be the reason why misconceptions about malaria have persisted.

The use of bed nets was deeply rooted within the culture, as participants had slept under bed nets even before they had known about malaria. This aligns with previous research, which has reported the habitual use of bed nets in this region [17, 18]. However, no participants reported using bed nets for any functions besides protection from mosquitoes. This contrasts with previous research from other communities along the Iquitol-Nauta road, where it was reported that people used nets as room dividers and for protection from spirits [18]. Additionally, participants in this study preferred LLINs to the traditional nets made from *tocuyo*, which is also a direct contrast with previous qualitative evidence from the same communities, which showed that participants had largely preferred the *tocuyo* nets in the past due to them offering more privacy and warmth [18, 19]. Participants were now aware that LLINs are more effective, and saw factors such as decreased privacy as something, which were necessary to get used to. However, some participants had chosen to use the *tocuyo* nets despite receiving an LLIN, due to fears of irritation caused by the insecticide, but these participants were unaware that these effects are transitory [38].

The overall preference for LLINs rather than *tocuyo* nets demonstrates that views on bed nets are changing due to increased awareness, and suggests that previous educational campaigns have been successful in facilitating change. However, some participants being unable to receive LLINs in the distribution scheme highlights the importance of going door to door, in order to maximize coverage. Many participants were also washing their LLINs more frequently than recommended by the WHO [22], which correlates with previous research which found that people from these communities often wash their treated nets too frequently [19]. Similar findings have also been reported in Sri Lanka [21]. This reduces the effectiveness of the net as it decreases the amount of insecticide within it [22]. Additionally, holes in the bed nets had not been repaired which is another factor decreasing their efficacy. WHO guidance specifies that LLIN distribution schemes should be accompanied with appropriate education about effective ITN use [39], however this is something which clearly has not been consistently implemented.

Furthermore, participants perceived mosquito biting times to be from 6 p.m. onwards, which confirms evidence of a shift in biting times of *An. darlingi* to earlier times [13–16, 40]. However, no participants were protected by their LLINs during this time. This dissonance between biting time and LLIN use also aligns with recent qualitative research conducted in San Juan [17], and is concerning as it demonstrates that this behavioural adaptation is allowing mosquitoes to evade LLIN protection.

Despite having perceived this change, participants have not adjusted their bed net use accordingly. This was attributed to lifestyle factors, which prevented them from using their nets earlier. However, this indicates that strategies which aim to persuade people to use their nets earlier are unlikely to be effective. Additionally, due to the plasticity of the *An. darlingi* [15], it is likely that it will further adapt in response to changes in net use to biting during daylight hours.

The socio-economic factors which are increasing the risk of malaria in the community should also be addressed, as these are counteracting the protective effects offered by LLIN use. Living in poor quality housing with no window or door screens facilitates the entry of mosquitoes and increases the vulnerability of the inhabitants [41]. This type of housing also causes spatial repellents, which were used by three participants, to be ineffective in reducing mosquito density as they permit free airflow [42]. The proximity of these houses to mosquito breeding sites also increases exposure to mosquitoes [43]. People are also left unprotected against these mosquitoes when not under bed nets due to inability to afford repellents. However, due to insufficient evidence that repellents are effective in preventing malaria, this is unlikely to be a contributing factor to the rising malaria incidence in the region [44].

The remoteness of these localities cause delays in accessing treatment as well as promoting the use of traditional herbal medicines. Traditional medicines are not recommended for malaria as there is insufficient evidence for their efficacy [45], and there is currently no evidence to suggest that *toronha* is effective in preventing or treating malaria. Use of traditional medicines, as well as distance from health facilities, is likely to be increasing transmission of malaria as well as the risk of mortality, as they prolong the duration of the illness.

It is clear that interventions are necessary to remove factors which are increasing malaria risk, and limiting the protection of bed nets. This need for additional intervention is also strongly felt by participants of the study. Regulated teaching should be provided in all communities, to ensure understanding of the disease before experiencing the illness. This is likely to dispel persisting misconceptions and decrease the use of natural medicines by making people aware that medical treatments are far more effective. This can be implemented by providing teaching programmes in schools in all communities, as this is recommended by the WHO as the most effective way to educate the most people [46]. Additionally, people receiving LLINs should receive appropriate education on effective net use during the distribution scheme, as recommended by the WHO [39], to stop incorrect maintenance of the LLIN from limiting their efficacy and to ensure that people are aware that irritant effects caused by insecticide are transitory.

It is also apparent that LLINs are not sufficient as malaria prevention long-term, due to the ability of *An. darlingi* to adapt its biting behaviour in response to net use. Despite participants requesting more frequent IRS, this unlikely to provide an effective solution due to *An. darlingi* having also adapted its behaviour to increased exophagy in response to IRS [15]. Long-lasting insecticide-treated hammocks (LLIHs) are proven to be effective for malaria prevention in areas where other vector control methods are insufficient [47], however the plasticity of the mosquito suggests that this will also not offer a permanent solution.

It is, therefore, necessary to shift the focus of malaria control efforts away from vector control. Interventions which focus on parasite control, which involve emphasis on active diagnosis using the rapid diagnostic test and prompt treatment of infected individuals, can act as a suitable alternative. This has been recommended by the Peruvian Ministry of Health (MoH) as a shift in strategy which is necessary to eliminate malaria in Loreto [6], and a similar strategy has previously been successful in eliminating malaria in Sri Lanka [48]. Treatment of asymptomatic individuals has been recommended as part of this change in strategy, which will be effective in reducing transmission by reducing the hypnozoite reservoir caused by *P. vvax* dominance. This can done administering mass primaquine treatment, which has been successful in the Maldives [49].

Efforts should also be made to overcome other factors which are increasing malaria risk in these communities. Treated netting can be used to cover gaps in housing which currently act as entry points for mosquitoes, and is likely to be cost-effective [50]. The number of mosquito breeding sites can be reduced by habitual larviciding, which is currently implemented in Peru to target breeding sites of the *Aedes aegypti* mosquito to prevent dengue [51], but this can also be used to target *Anopheles* breeding sites which can also be successfully larvicided [52]. Additionally, the introduction of mobile health centres in remote communities—which have been successfully implemented in Sri Lanka [48]—will prevent delays in seeking medical treatment.

These recommendations are not without cost and it may be difficult to implement them all, especially in a country with a restricted budget. Funding from malaria control interventions in Peru comes primarily from the Peruvian government, which spends only $0.1 per person at risk of malaria [3]. It is likely that an increase in budget from the government will have a positive impact on malaria cases in Peru if spent on evidence-based interventions. Nonetheless it is important that the financial implications of what an ideal malaria programme would involve should be considered against the current financial climate as well as demands from other sectors, however this issue is beyond the scope of this paper.

### Strengths and limitations

This research was conducted by a researcher who was not native to Peru. This allowed the analysis to be conducted from a purely objective standpoint where findings were compared only with literature and guidance regarding vector control, and not with regional customs. However, this may also have caused participants to see the researcher as an ‘outsider’ and may not have been as forthcoming with information as a result of this.

A translator was used for the interviews, which breaks the interview exchange and impedes the free expression of ideas from the participant. There is also always a risk of loss of meaning through translation. However this translator had conducted qualitative interviews 1 year previously and was familiar with the interview process, which helped to mitigate this limitation. Additionally, the translator was native to Loreto, which may have made the participant feel more comfortable as they are giving answers to someone who is familiar with their culture. This also alleviates the drawback of the researcher not being native to Peru.

Additionally, these results are likely to be generalizable to rural populations in Loreto, however not urban populations as all 5 localities where the study was conducted were rural.

## Conclusion

This research establishes that there are many factors which can currently be attributed to the rising malaria incidence in Loreto, and that despite widespread uptake of LLINs in distribution schemes their use does not offer adequate long-term protection against malaria in this region. Findings confirm that the recent behavioural adaption of *An. darlingi* is allowing it to evade bed net protection, due to a dissonance between new mosquito biting times and timings of bed net use. This study also adds to previous qualitative literature regarding bed net use in Loreto, and demonstrates changing perceptions due to increased awareness about malaria.

These findings can be used to influence future policy changes, which are necessary to eliminate malaria in Peru. It is also beneficial to conduct research on any further adaption of *Anopheles darlingi* in response to changes in policy.

## Data Availability

The datasets used and/or analysed during the current study are available from the corresponding author on reasonable request.

## Appendices

### Appendix A: Localities

**Table:**

Locality	Name	Participants
1	Zungarococha	P01, P02, P03, P04, P05
2	El Dorado	P06, P07, P08, P09
3	Rosa Mystica	P10, P11, P12, P13
4	Pujil	P14, P15, P16
5	Ex Petrolieros Zone 1	P17, P18, P19, P20

### Appendix B: Topic guide

#### Preceding each interview

1. Introduce researcher and translator.
2. Explain study using the participant information sheet.
3. Obtain written consent for the interview process and bed net observation
4. Ask name and age before commencing the interview.

**Table:**

Topic	Questions and Probes
Knowledge about malaria and bed nets	1. What do you know about malaria? *Probe: How seriously do you view the illness*? *Probe: Have you had experience with the illness?* 2. How important do you think it is to prevent malaria? *Probe: Do you do anything to prevent malaria?* 3. What do you know about bed nets? *Probe: Do you think they are important?* 4. Do you know about the different types of bed net? *Probe: traditional or insecticide-treated?*
Personal attitudes/opinions towards bed nets	1) How long have you been using bed nets? 2) Did you receive a bed net by the donation scheme? 3) What do you think of your new bed net? *Probe: How do you feel sleeping under it?* 4) Do you have a preference between your old net and your net net? *Probe: Did you prefer sleeping under a traditional or a LLIN?* 1) What do you think about the net distribution schemes? 2) Have your views on bed nets changed over time?
Bed net practice	1) How do you maintain your bed net? *Probe: Repair? Washing*? 2) Is the net tucked in during use? (i) What are your sleeping arrangements? *Probe: How many people under one net?* (ii) From what times do you use your bed net? *Probe: Time you go to bed?* *If after 6: What are you doing before bed?*
Community views and practices	(i) Do you think that other people here have the same view as you? (ii) Can you tell me about other ways that malaria is prevented in the community? *Probe: Rapid diagnostic testing*, *IRS* (iii) Do you think the government should do more to prevent Malaria?

#### Following each interview

1. Ask if there is anything else the participant would like to talk about relating to malaria or bed net use.
2. Thank them for participating in the study.
3. Make sure the participant has the participation sheet containing contact details, and that they understand that they are able to contact the researcher with anything relating to the study.

## Abbreviations

WHO: World Health Organization
LLINs: long-lasting insecticidal nets
MoH: Ministry of Health
